# Contextual effects on health: systematic review of studies using natural experiments among migrants

**DOI:** 10.1093/eurpub/ckac131.212

**Published:** 2022-10-25

**Authors:** L Biddle, M Hintermeier, D Costa, Z Wasko, K Bozorgmehr

**Affiliations:** 1 Population Medicine and Health Services Research, University Bielefeld, Bielefeld, Germany; 2 Section Health Equity Studies and Migration, University Hospital Heidelberg, Heidelberg, Germany

## Abstract

**Background:**

Many studies on contextual health effects suffer from compositional bias and selective migration into neighbourhoods. Longitudinal natural experiments have the potential to overcome these limitations, and there are several opportunities for this research design in the migration context. We aimed to synthesize evidence from natural experiments among migrants studying the effect of contextual factors on health and healthcare.

**Methods:**

Peer-reviewed literature in English or German was systematically searched in four major databases in December 2021. Following systematic abstract- and fulltext-screening, 32 studies were included for analysis. Evidence on contextual impacts on physical and mental health, mortality, and healthcare was narratively synthesized and quality appraisal conducted.

**Results:**

We found four types of contextual health effects: factors of the place of residence in receiving countries (n = 6), migration-context interactions (n = 10), policy environments (n = 15) and cultural factors (n = 1). Results show the negative impacts of post-migratory contexts on physical health and mortality and the favourable impacts on child health. Impacts on mental health are mixed. Analyses of policy contexts indicate the negative impacts of restrictive migration and social policies on healthcare utilization, mental health and mortality as well as the positive effects when restrictions are lifted.

**Conclusions:**

Natural experiments can serve as powerful tools in disentangling the effect of context on health and reducing bias through self-selection. Results demonstrate the negative impacts for health which lie at the nexus of migration and neighbourhood disadvantage. At the same time, studies uncover the potential of health, welfare and visa programs to counteract such disadvantages and create healthy post-migratory contexts. With careful consideration of causal pathways, results from migration contexts can serve as a magnifying glass for effects of context in other population groups.

**Key messages:**

• Natural experiments can serve as powerful tools in disentangling the effect of context on health and reduce bias through self-selection.

• Results show the negative impacts for health that lie at the nexus of migration and neighborhood disadvantage, as well as the potential of inclusionary policies to counteract them.

